# Role of contrast-enhanced ultrasound in evaluation of the bowel

**DOI:** 10.1007/s00261-017-1399-6

**Published:** 2017-12-19

**Authors:** Alexandra Medellin, Christina Merrill, Stephanie R. Wilson

**Affiliations:** 1 Department of Radiology, University of Calgary, Foothills Medical Centre, 1403 29 St. NW, Calgary, AB T2N 2T9 Canada; 2 Sonographer, Foothills Medical Centre, 1403 29 St. NW, Calgary, AB T2N 2T9 Canada; 3 Department of Medicine, Division of Gastroenterology, University of Calgary, Foothills Medical Centre, 1403 29 St. NW, Calgary, AB T2N 2T9 Canada

**Keywords:** Contrast enhanced ultrasound, Ultrasound, Bowel, Inflammatory bowel disease, Gastrointestinal

## Abstract

Contrast-enhanced ultrasound (CEUS) of the gastrointestinal tract provides vascular information helpful for characterizing masses and other pathologies in and around the bowel, similar to contrast applications in other solid organs. However, the use of microbubble contrast agents for the bowel provides additional unique contributions as it gives both subjective and objective information about mural and mesenteric blood flow, invaluable for the determination of disease activity in those many patients affected by inflammatory bowel disease (IBD). IBD is a lifelong chronic disease and has its peak age of onset in adolescence or young adult life. Today, we have moved away from treating patient’s symptoms and strive instead to alter the course of disease by obtaining mucosal healing. Expensive and aggressive biologic therapies and lack of agreement of patient’s symptoms with their disease activity and complications necessitate frequent imaging surveillance, which must be safe, readily available, inexpensive, and effective. Ultrasound with the benefit of contrast enhancement meets these requirements and is shown in meta-analysis to be equivalent to CT and MRI scans for these indications.

**Electronic supplementary material:**

The online version of this article (10.1007/s00261-017-1399-6) contains supplementary material, which is available to authorized users.

Sonographic evaluation of the bowel has become one of the most valuable advancements of ultrasound in the past decade especially for patients with inflammatory bowel disease (IBD) who require frequent monitoring of their inflammatory activity due to its natural fluctuating course and the current use of biologic therapies [1]. Multiple studies have shown the accuracy of grayscale ultrasound (US) for determination of disease extent and activity [1–5]. Although, bowel wall thickness is the most reliable feature reflecting inflammation in the bowel wall, some patients with quiescent disease continue to show bowel wall thickening in the absence of active inflammation [5–7]. In addition, sensitive detection of response to therapy may require more than just gross morphologic assessment. Therefore, detection and evaluation of vascular flow is an important factor in the diagnosis of bowel disease.

It is known that active inflammation is accompanied by neoangiogenesis of the bowel wall and vascularization of the adjacent mesenterium [8–10]. Mural blood flow detected with color Doppler imaging (CDI) has been viewed for many years as a reflection of active inflammation, allowing for monitoring of disease activity [9, 11]. On the other hand, if color Doppler signal is absent, this may suggest inactive disease in the case of IBD [12] or ischemia in the setting of acute or chronic abdominal pain [13].

Current state of the art ultrasound equipment provides sensitive Doppler technology which can show mural and mesenteric blood flow with relative ease [11, 14]. However, CDI reflects large blood vessels with fast flow [11, 12, 15]. Signals may be difficult to detect in large patients, in those with inherently weak Doppler signals, and in bowel loops located deep within the abdomen or pelvis. However, the most significant weakness of CDI is its inability to show blood flow at the capillary or perfusion level, important for determination of disease activity [14]. Therefore, a confident evaluation of the bowel with only grayscale ultrasound and CDI may be deficient.

As with other cross-sectional modalities, the introduction of contrast agents for ultrasound has improved the assessment of the bowel vasculature. CEUS is performed with microbubble contrast agents which provide subjective and objective parameters that reflect inflammation by showing enhancement of the bowel wall and mesentery. Studies have shown a direct correlation between level of bowel wall enhancement and active inflammatory disease on pathology [16–19]. In addition, they enable the creation of time–intensity curves from which quantification of bowel wall enhancement will reflect the bowel fractional blood volume, the blood flow, and the transit time. Based on this quantification analysis, the presence and degree of inflammation can be objectively assessed and followed over time. This is particularly important for evaluation of treatment response by comparing quantification values over a series of studies.

Additionally, CEUS can be used for characterization of masses in the bowel wall or adjacent mesenterium. This resolves dilemmas which frequently arise during grayscale evaluation as colonic content, inflammatory polyp, and neoplasm can all overlap in appearance. Further, CEUS is particularly important in the differentiation of inflammatory masses as either phlegmon or abscess, as their treatment would vary.

In our experience, the addition of CEUS for assessment of bowel pathology has significantly improved our ability to detect, evaluate and objectively quantify the presence of bowel inflammation and its complications. The non-ionizing nature of this technique makes ultrasound a desirable alternative in the frequently young patient with IBD who has a high demand for imaging throughout the chronic course of their disease. This study highlights the performance of CEUS, its bowel indications, and contributions to patient management.

## CEUS technique

Sonographic evaluation of the bowel begins with identification of abnormal loops and determination of disease extent and location. US features of active disease include bowel wall thickening (> 3 mm for small bowel and > 5 mm for colon), hyperemia detected with CDI [11], the presence and amount of inflammatory fat and lymphadenopathy [18]. These parameters allow for the selection of the most abnormal segment that can be studied with CEUS. Two important considerations when selecting the bowel segment for evaluation with CEUS include a well-visualized loop of bowel within the field of view with minimal or no peristalsis and bowel wall thickness of > 4 mm. This will ensure the best technical acquisition, especially for quantification. In our experience, loops in the lower quadrants, away from the influence of respiratory excursion, are optimal. In the majority of the cases, this is possible, as IBD most often affects the terminal ileum and the cecum. If an abnormal loop is identified with significant peristalsis, 20 mg IV of hyoscine butylbromide (Buscopan) over 1 min or glucagon 1 mg IV is used to minimize the bowel activity, if there are no contraindications. In our experience, antimotility medications are required in less than 10% of the cases of bowel CEUS.

### Microbubble contrast agents

CEUS is performed on a fasting patient, using a microbubble contrast agent that enhances the ultrasound signals and allows for real-time evaluation of the vascularity [20]. In Canada, our experience has been with perflutren microspheres, marketed as Definity (Lantheus Medical Imaging, Billerica, MA) [18]. Sulfur hexafluoride, marketed as Sonovue (Bracco, Italy) in Canada, Europe, and Asia and Lumason in USA, is a similar agent with equivalent performance. These contrast-enhancing agents are gas-filled microbubbles with a phospholipid shell and are administered to the patient as a bolus intravenous injection [18, 21]. Microbubbles remain within the capillaries throughout the examination and they oscillate in the bloodstream in response to the application of an ultrasound field, producing non-linear harmonic frequencies that are detected at CEUS [22]. Our standard volume of contrast agent for a bowel quantification study is generally twice the standard dose for liver mass evaluation: with perflutren lipid microspheres (Definity, Lantheus Medical Imaging) 0.4 ml, and with sulfur hexafluoride (Lumason, Bracco Diagnostics Inc) 4.8 ml, both followed by a 5 ml saline flush.

### Machines and probes

The selected bowel is imaged with a high-resolution probe, in the frequency range of 5–9 MHz, using contrast-specific imaging techniques that currently are available on most high-end ultrasound systems. We choose the frequency optimal for the bowel evaluation which on our equipment is a C9-2 (EPIQ, Philips Bothell, WA) with a lower frequency reserved for occasional very large patients with deep bowel. Endovaginal probes with contrast capability also work exceptionally well to study deep ileal loops, in particular. Linear probes and high-resolution convex probes are also highly successful for evaluation of the perineum and anal canal.

Contrast imagining technique parameters include a low mechanical index and contrast-specific subtraction, whereby background tissue echoes are removed from the image so that a microbubble-only image is created.

Standard CEUS settings are contrast gain of 50–60%, mechanical index of 0.05, dynamic range of 40 dB, and focal zone set at maximal depth. We maintain the same parameters between examinations for standardization of the procedure. Obese patients and deeper bowel segments will reduce visualization and signal intensity; therefore, grayscale optimization is an important factor when performing bowel studies.

Although most US systems do allow for CEUS of the bowel and also generation of time–intensity quantification curves (TIC), there is considerable variability in the output of the machines which hampers the progress of this exciting technique. Additionally, many workstations, for quantification of blood flow, are machine/vendor specific which is additionally limiting. In our institution, we perform all of our quantifications with Q-Station (Philips Healthcare Ultrasound, Bothell WA). However, a more versatile system is VueBox (Bracco, Geneva, Switzerland) which is able to analyze data for quantification from all US systems following entry of 2D DICOM datasets provided by the vendors to Bracco.

### Exam performance

Patients generally lie comfortably in a supine position. Quiet respiration is encouraged. To perform CEUS, the transducer is placed at the region of interest (ROI) selected on the initial grayscale assessment. The abnormal bowel segment is evaluated in a sagittal orientation to minimize out-of-plane effects of respiratory motion and to facilitate generation of quality TIC allowing for quantification analysis. After contrast is injected through the patient’s IV, a continuous acquisition is initiated, even before the arrival of the first bubble in the field of view, and lasting for 2 min with no motion of the transducer. Most ultrasound equipment will allow for initial evaluation of the quantification curves at the bedside to assess technical quality of the time–intensity curve and to obtain preliminary results. If CEUS fails, or is technically inadequate, a second contrast injection may be performed, after a several minute delay.

### Subjective assessment

**Fig. 1:**
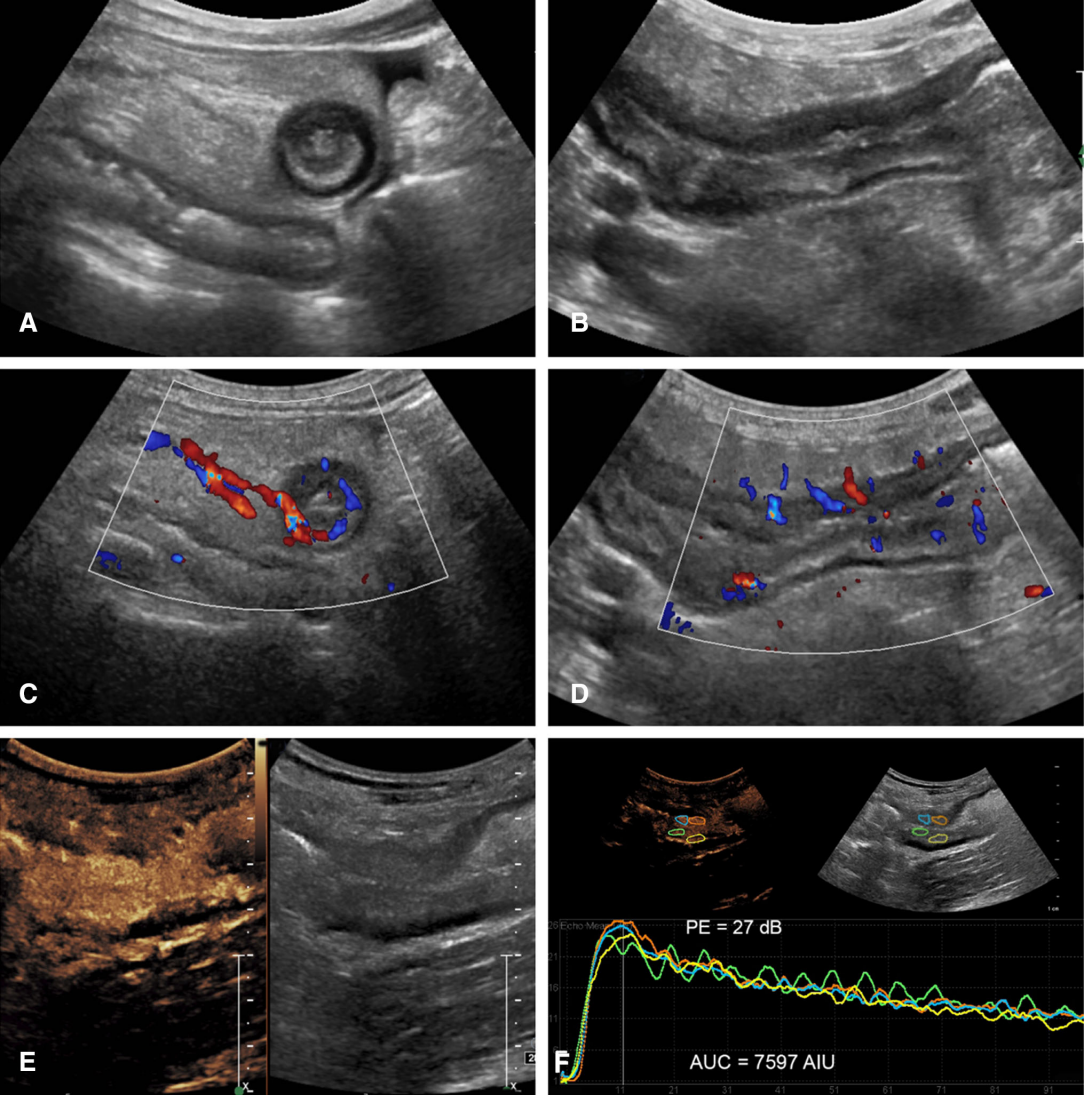
A 41-year-old female with severe active Crohn’s disease of a long segment of the neoterminal ileum. **A** Axial **B** and sagittal images of a thickened segment with a large amount of surrounding echogenic inflammatory fat and trace of free fluid. Wall thickness is 1 cm with some loss of the bowel layers. **C** Axial and **D** Sagittal color Doppler images show significant wall vasculature and prominent straight mesenteric vessels (a color Comb sign). **E** Dual-screen CEUS display of the abnormal segment shows transmural hyperenhancement on the left, and a low MI grayscale image for reference on the right. **F** Generated time–intensity curves from 4 ROIs placed on the anterior and posterior bowel walls (colored circles) show similar curves with high-enhancement values (PE 26 dB) during wash-in followed by very slow progressive decline of contrast enhancement overtime with a large area under the curve. The curves generated from the anterior and posterior wall are essentially equivalent

Subjective evaluation of CEUS includes assessment of the degree and pattern of mural and mesenteric enhancement [23, 24]. Generally, inflamed bowel shows transmural enhancement with rapid enhancement of the bowel wall to a higher level than the adjacent tissue and in proportion to the level of inflammation, initially leading to a peak intensity before declining due to the excretion and distribution of the contrast agent [21, 22] (Fig. 1 and Supplementary Video 1). Generally, enhancement is transmural although less often, only the submucosal layer may be involved.

With experience, observation of the wash-in and decline of contrast agent in the bowel wall may be interpreted as reflective of mild disease with low peak and rapid decline and of more severe disease with a higher peak intensity and longer duration of enhancement.

**Fig. 2:**
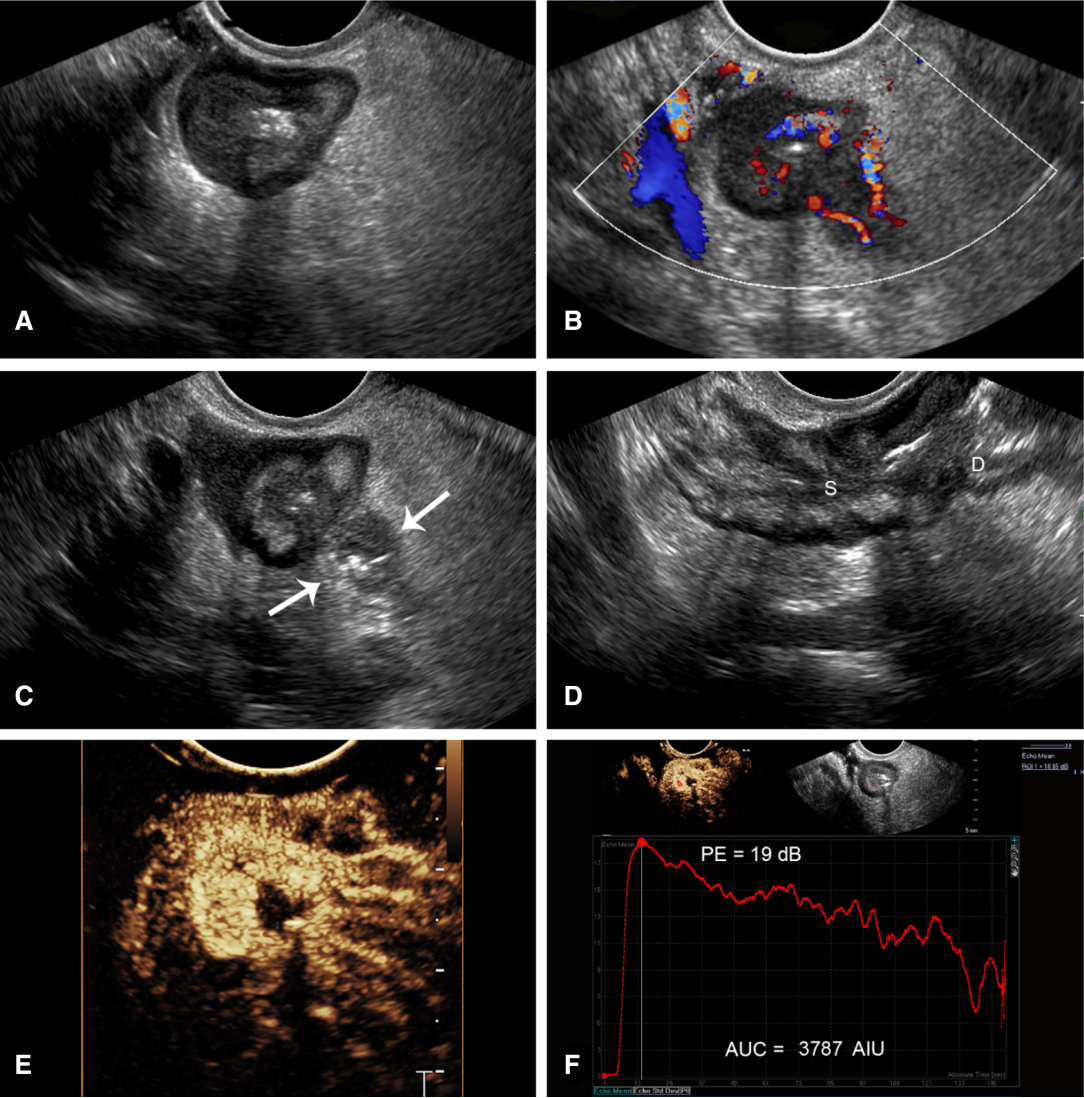
Capability of endovaginal scan to show classic features and complications of CD, plus performance of CEUS. A 23-year-old female with Crohn’s disease for 11 years. **A** Axial B-mode endovaginal image of the terminal ileum deep in the pelvis shows wall thickening of 7 mm with loss of the wall layers. **B** CDI shows mural hyperemia with prominent mesenteric vessels. **C** Axial grayscale shows localized perforation and adjacent abscess (arrows) (Supplementary Video 3). **D** Sagittal view shows a stricture (S) with luminal apposition and prestenotic dilatation (**D**) of the proximal bowel reflecting mechanical bowel obstruction. **E** CEUS shows transmural hyperenhancement and comb sign (Supplementary Video 2). **F** Top left, ROI (red circle) placed in the abnormal loop. Top right, corresponding grayscale. Bottom, TIC generated from CEUS showing rapid rise of contrast, high peak of 19 dB and AUC of 3787 consistent with moderate activity

Additionally, the vascularization of the mesentery can be evaluated subjectively by demonstration of a comb sign (Fig. 2E and Supplementary Video 2), named after its more familiar appearance on CT scan and representing the filling of prominent straight intestinal arterial branches in the mesenteric arcade. This is seen invariably with active disease.

In cases where the abnormal loop of bowel is deep within the pelvis, endovaginal B Mode and CEUS can be performed (Fig. 2). This excellent technique allows for close evaluation of the bowel with high-resolution US probes with great definition of wall abnormalities, in addition to superb capability to show blood flow and complications (Supplementary Videos 2 and 3). CEUS can also be performed using the endovaginal approach with creation of quantification curves (Fig. 2F).

**Fig. 3:**
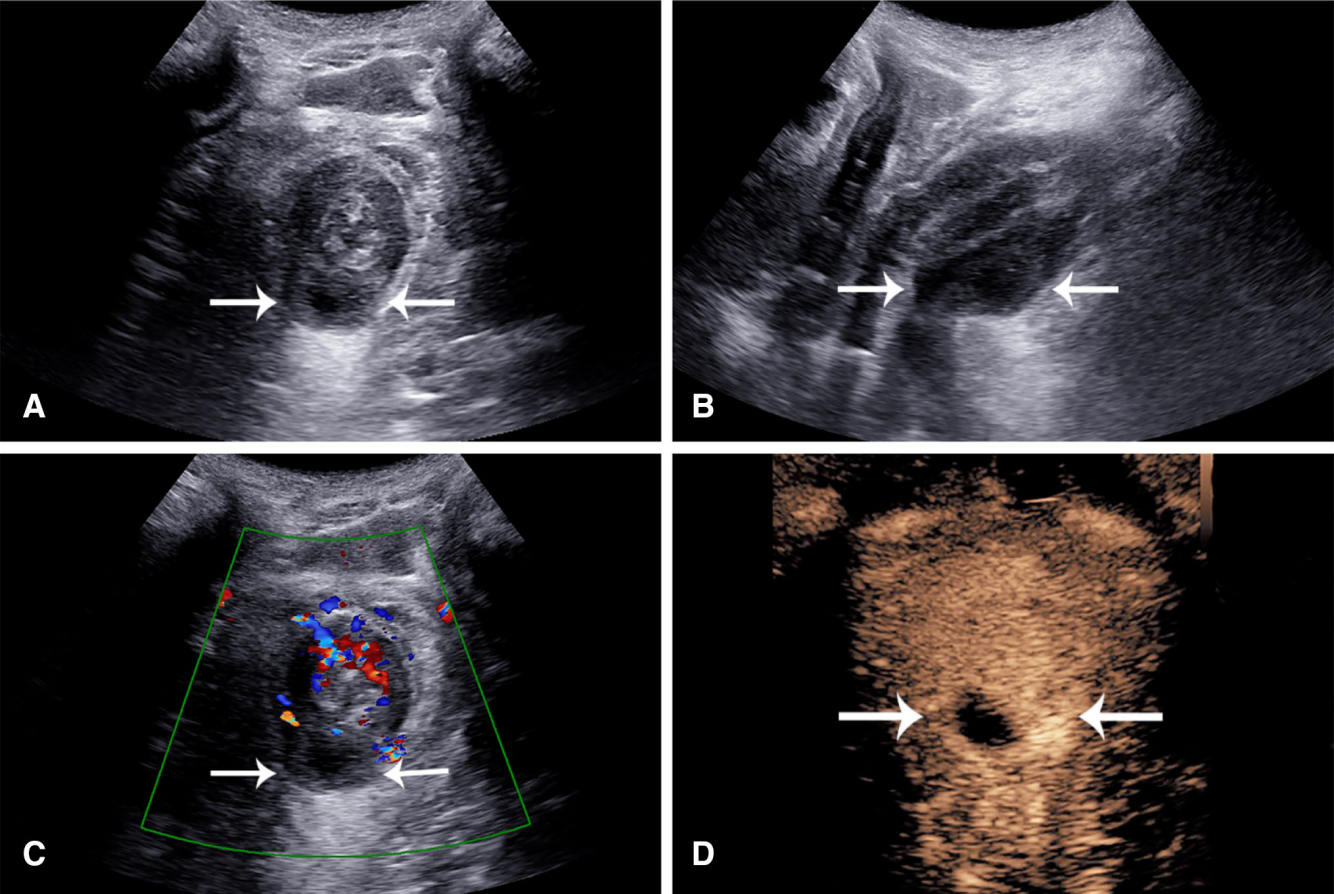
A 53-year-old male with CD for one year with very few symptoms. **A** Axial and **B** sagittal grayscale perianal scan shows a perianal intramural hypoechoic mass involving the posterior anal canal from 6 to 8 o’clock (arrows). **C** Color Doppler of the same region shows hypervascularity of the anal canal and no color signal within the mass. **D** CEUS shows peripheral enhancement of the mass with a central avascular component suggesting pus, consistent with an abscess cavity (Supplementary Video 4)

Further, perianal disease and evaluation of perianal complications are well performed with US and CEUS from a perianal approach (Fig. 3 Supplementary Video 4). The use of CEUS with this technique markedly improves differentiation of phlegmon from perianal abscess.

### Objective assessment

An advantage of CEUS is the generation of time–intensity curves as an objective parameter of bowel enhancement. The recorded TICs can be analyzed using built in software on the ultrasound machine or using remote workstations. The raw native data from the 2-min Cine-clip stored in the US system are log compressed and exported in DICOM format to a PACS workstation or other analysis system, where the data are re-linearized. From these data, the linear TICs with appropriate curve fitting are generated allowing for measurement of blood flow parameters within the bowel wall.

**Fig. 4:**
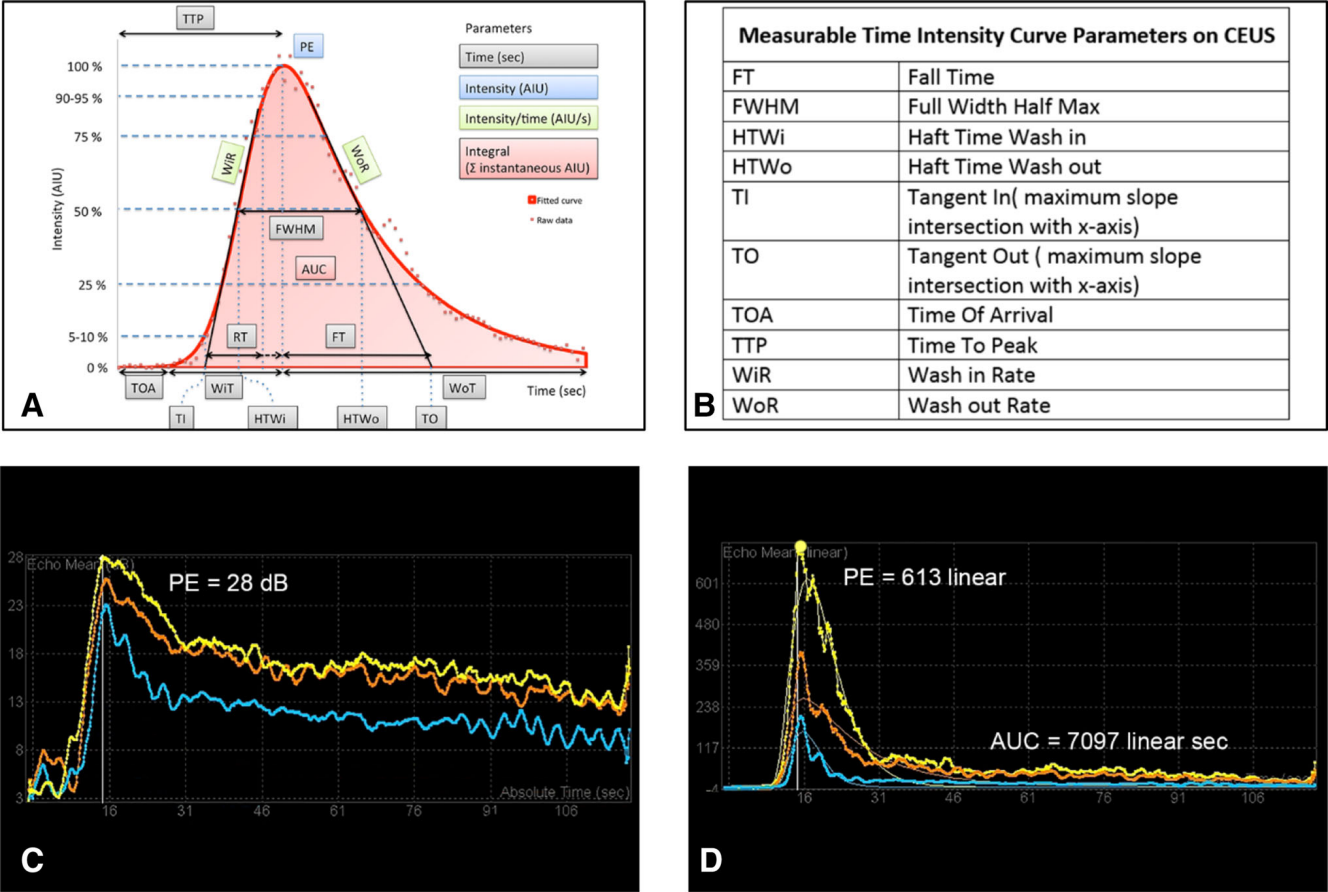
Time–intensity curves for determination of disease activity. **A** Schematic representation of a time–intensity curve of the bowel. **B** Shows the key to the abbreviations shown on the schematic in **A**. **C** Linear data with curve fitting and **D** logarithmic TICs from a 52-year-old male with CD. The patient has severe disease with a PE of 28 dB and an AUC of 7097 linear sec (AIU). Use of multiple regions of interest is a quality measure to ensure accuracy of data and explains the three demonstrated curves. Some variations between the curves are evident but do not change the degree of activity (severe > 23 dB). **A** and **B** are reproduced with permission from contrast-enhanced ultrasound 2nd Ed [26]

The TIC is created by placing a ROI within the enhanced bowel wall (Supplementary Video 5). In our institution, we draw free hand at least three ROIs (~ 1 cm^2^) centered in the most enhanced segment, and only including the bowel wall. The placement of the ROI depends on technical factors. Most often it is easiest to place the ROI in the anterior wall if luminal gas is present but our own unpublished reproducibility data show that placement within a visibly uniformly enhancing portion of the bowel wall, either anterior or posterior, is suitable, (Fig. 1F) This is supported by Greis C who shows a direct correlation of Peak Enhancement and concentration of microbubbles [25]. The ROI should not overlap and its size can be also standardized by software. From this, an enhancement curve is created based on the wash-in and wash-out characteristics. This curve shows the wash-in slope, time to peak, peak intensity, area under the curve (AUC), mean transit time, time from peak to ½, and rise time, all curved fitted and presented in log-normal (Fig. 4A, 4B) [26]. We draw three regions of interest to confirm reliability of the curve and its measurements. As there is a lot of information associated with a 2- or 3-min acquisition of data for TIC generation, acquiring information from a single ROI might introduce error from peristalsis or other movement, for example. The use of three non-overlapping ROIs will detect if one or more datasets are unreliable and ensures higher standard of results. From these three ROIs, we select the linear curve allowing for the best curve fitting for the final result.

**Fig. 5:**
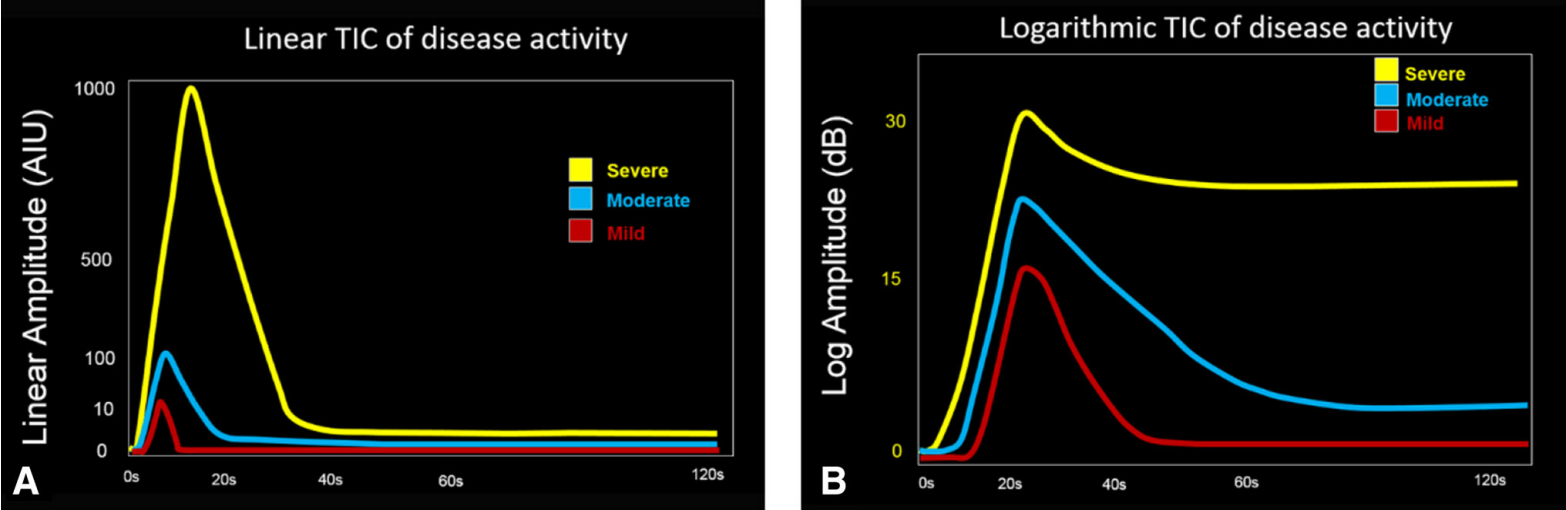
Schematic representation of time–intensity curves (TIC) and disease activity. **A** Linear and **B** logarithmic TICS are shown for mild (red), moderate (blue), and severe (yellow) disease activity. The linear curves generally resemble each other showing a peak above the baseline with a rapid rise and a fast decline. The logarithmic curves, by comparison, show variation in the signal intensity and also the rate and amount of decline. The more manageable scale on the log TIC makes it easily useable. Thresholds for disease activity on CEUS include values in the range from 10 to 30 dB, a highly useable range, which encompasses linear values from 10 to 1000, making their clinical use a challenge

Most analytic software programs offer two types of graphic display for viewing a TIC, log and linear representations. Although the linear graphs show the most realistic presentation of the raw data, allowing for the most accurate and precise measurement of inflammatory parameters (Fig. 4D), its large range of numerical values makes it difficult for presentation of data that would be of a reasonable clinical use. For example, a value of 100 in the linear display corresponds to 20 dB logarithmic display and 1000 linear would be 30 dB. Therefore, we view a log display for measurement of peak enhancement in dB only, as it makes a range of values which are more manageable and compatible with classification ranges of disease activity based on the bowel wall thickness for clinical use (Fig. 4C) [20]. Our own published data show that the clinical values for peak enhancement range between 10 dB and 30 dB and encompass patients with quiescent disease at the lower spectrum, less than 15 dB, and those with severe active inflammation in the order of 23 dB or greater, with more moderate inflammation showing values in between (Fig. 5) [18]. Additionally, following treatment, a change of the peak enhancement reflects a real change, whereas using linear data, huge differences in the values may reflect virtually no significant change at all.

Our software analysis system generates a printout for every patient including all of the parameters listed above, with the peak enhancement in dB and all other parameters generated from the curve of the log-normal linear data. To this date, we predominantly utilize the peak enhancement in dB, the AUC, measured in arbitrary intensity units, and the time to peak, in seconds for establishing an initial baseline activity assessment and to show changes over time with treatment or with natural disease progression (Fig. 4C, D). Subjective and objective observations of the TICs show that with severe disease, the PE is high and the decline may be delayed and at a higher enhancement level (yellow curve, Fig. 5B). On the other hand, with mild disease the PE enhancement is low and the decline is rapid and more closely reaches the baseline (red curve, Fig. 5B). These same assessments are more difficult to recognize when looking at the linear curves (Fig. 5A).

In centers in Europe, CEUS quantification analysis is often expressed as a percentage change of peak enhancement and of the AUC before and after treatment [21, 27–29]. In the study by Quaia [30], two ROIs are drawn, one in the thickest part of the anterior wall or covering the entire bowel wall in cases when the lumen was not visible and the second as an internal reference at the adjacent mesentery. TICs are also fitted with a non-linear least-squares regression method to a proprietary log-normal model with assessment of multiple curve parameters [30, 31]. These studies only compare exams between the same patient over time.

We prefer, instead, that this robust technique allows for integration of CEUS parameters with other established subjective and objective measures of disease activity, especially wall thickness and signal on color Doppler imaging, [32] to allow for categorization of disease between large numbers of patients and for more easily applied determinations of disease activity [18]. In addition, it is invaluable for follow-up of patients over time.

Today, there is considerable variation in the output of US equipment for quantification techniques. This inconsistency in display makes acceptance of this technique for routine clinical applications a challenge and it is hoped that standardization of methods, displays, and reporting will occur in the near future as it has for other imaging techniques over time.

## Indications for CEUS of the bowel

### Disease activity

Determination of disease activity is most important when evaluating bowel inflammation. This is essential at the time of new diagnosis, during surveillance, to monitor treatment response and for the total assessment of symptomatic patients.

Evaluation of disease activity is performed in our institution using an ultrasound global assessment based on objective measurement of the bowel wall thickness and semiquantitative assessment of the inflammatory fat and signals on CDI [11, 18]. Wall thickness is considered as the strongest predictor of disease activity followed by hyperemia on CDI, as shown by multiple prior studies [11, 32–37] and in a recent publication by Novak, et al. [32]. CEUS has a good direct correlation between the level of PE and degree of bowel wall thickness for assessment of disease activity [18]. Observations are classified in four categories, according to the severity of the findings shown: normal/remission, mild, moderate, or severe disease. If the bowel wall is more than 4 mm in thickness, CEUS may be performed to confirm grayscale findings and to record baseline parameters for use if there is a treatment change or for future reference. CEUS parameters are now integrated within our global assessment as an additional objective parameter of disease activity (Table 1). This allows for a more systematic approach to better follow therapeutic change. CEUS is now widely accepted and recommended in inflammatory bowel disease in the European Federation of societies for Ultrasound in Medicine and Biology (EFSUMB) guidelines [38].

**Table 1:** USGA with integration of CEUS

Performance of Ultrasound Global Assessment				
Ultrasound features of activity	Classification			
	INACTIVE	MILD	MODERATE	SEVERE
Wall thickness (mm)	< 4	4.0–6.0	6.1–8.0	> 8
Color Doppler imaging (CDI)	Absent	Small regions of color No vessel structure	Medium vessel length	Circumferential vessels ± Mesenteric vessels
Inflammatory fat	Perienteric region resembles normal mesenteric fat	Masslike Slightly echogenic LESS AREA than the bowel on axial view	Masslike More echogenic EQUAL AREA to the bowel on axial view	Masslike Significantly echogenic GREATER AREA than bowel on axial view
*With integration of contrast-enhanced ultrasound (CEUS)—peak enhancement*				
CEUS PE (dB)	Absent—15 dB	15–18 dB	18–23 dB	> 23 dB

Reproduced with permission from Ref. [9]. The ultrasound features assessed for disease activity are listed in the left column. The observations are classified from inactive through mild and moderate to severe inflammation on the basis of the observations listed under each. The integration of CEUS PE in decibels is listed on the bottom row

### Indeterminate cases

**Fig. 6:**
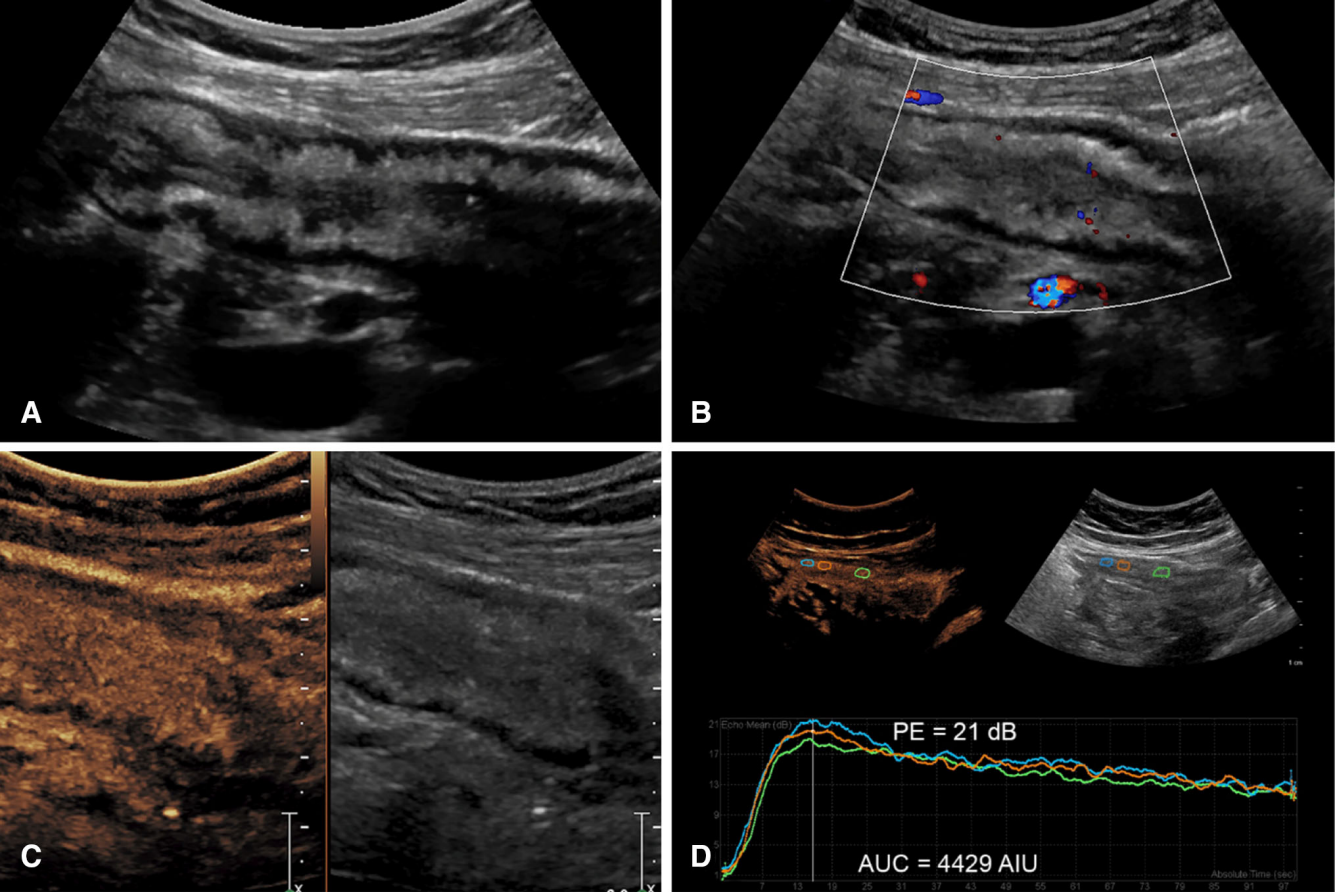
Indeterminate activity assessment resolved by CEUS in a 53-year-old female with Crohn’s. **A** Sagittal grayscale image shows moderate wall thickening of 7 mm. **B** CDI of the abnormal ileum does not show any color Doppler flow. Activity is therefore indeterminate. **C** CEUS shows transmural hyperenhancement. **D** CEUS and generated curve confirm moderate activity as suggested in image **A** and **C**. ROIs (color circles) are placed in the anterior bowel wall

Both wall thickness and hyperemia of the bowel wall when present are good predictors of disease activity [3, 18]. However, inactive disease can be present with normal bowel wall thickness or with persistently abnormal bowel wall thickening. Differentiation of active from inactive disease then relies on detection of increased vascularity within the bowel wall. This is initially performed with color Doppler, where inactivity will show little or no blood flow. However, in some cases CDI is not reliable due to patient body habitus or technical factors. In these instances, failure to show color Doppler signal within a thickened and abnormal appearing loop of bowel poses a dilemma between inactive disease or merely a technical failure resulting in poor signal detection. Therefore, activity is indeterminate. In this circumstance, CEUS is an invaluable tool often showing transmural enhancement and high CEUS parameters in cases of active disease where no color signal can be detected at all due to technical failure (Fig. 6, Supplementary Video 6) [39]. On the other hand, in patients with very long standing disease where fat infiltration of the submucosal layer often creates thickened bowel wall with no color Doppler, chronic/quiescent disease needs to be differentiated from acute on chronic active disease.

### Differentiation of strictures in IBD

Ultrasound is long recognized as a very reliable technique for the detection of strictures and associated incomplete mechanical bowel obstruction. This dynamic real-time technique shows dilated prestenotic segments and also evidence of dysfunctional peristalsis. Differentiation of strictures poses a frequent dilemma for imagers. This can be easily resolved by the addition of subjective and objective evaluation of the bowel blood flow with CEUS by showing the contribution of inflammation to the stricture and may be aided further by stiffness determination with elastography. Strictures in IBD may be inflammatory or chronic in nature. Differentiation of these two broad categories is recognized as a major role for imaging techniques as management differs. Several investigators have addressed the role of CEUS [40–42] and also elastography to characterize these strictures with ultrasound [42, 43]. Our own study shows that, in patients with chronic stenotic disease, smooth muscle hyperplasia/hypertrophy may contribute more to stricture than fibrosis [44] similar that to prior study by Baumgart [45]. Additionally, we show an inverse relationship between velocity measurements on shear wave elastography and peak enhancement on CEUS. [30]. Quaiai et al. show differentiation of inflammatory vs fibrotic strictures in which determination of active inflammation would favor the role of medical therapy [40].

### Monitoring response to therapy

Medical therapy for patients with inflammatory bowel disease has evolved from the treatment of patient’s symptoms and complications to active efforts to modify the natural course of the disease by achieving mucosal healing as shown at endoscopy. Today, the use of long-term biologic therapies has changed the imaging management of this patient population with recognition that interval surveillance is a requisite component of treatment management. Additionally, medical therapy with steroids, immune modulators, and biologics all necessitate imaging surveillance to assess for treatment response and detection of complications in these immunosuppressed patients. Various publications have shown that the use of CEUS with TIC analysis can differentiate pharmacologic therapy response and identify non-responders [29, 30]. This distinction is very important for patient outcome and prognosis. Early recognition of inadequate treatment response would alleviate a long, expensive, and ineffective treatment [46].

Therapy response can be categorized as showing complete positive response, no response, or various grades of partial response. We do this grading by evaluating all parameters shown on grayscale and also on CEUS. While a complete positive response may lead to virtual resolution of all observations, more often a partial response may only show reduction of the CEUS parameters with the grayscale and CDI parameters continuing to suggest active disease. CEUS with grayscale evaluation may also show complete lack of response suggesting the necessity of dose escalation or change of therapeutic regime. Surveillance scans are performed of all patients on biologic therapy as inflammatory parameters are recognized as unreliable determinants of disease.

## Additional uses of CEUS in bowel disease

### Inflammatory masses

In IBD and other bowel conditions, there is a potential for development of inflammatory masses representing fluid containing abscesses or phlegmons, where there may be severe inflammation but no actual drainable pus. Differentiation of these masses on grayscale US is often challenging as there is a strong tendency for overlap in their US imaging appearance with all frequently showing as a hypoechoic mass. This distinction is of extreme clinical significance, as management, prognosis, and therapy differ [47].

**Fig. 7:**
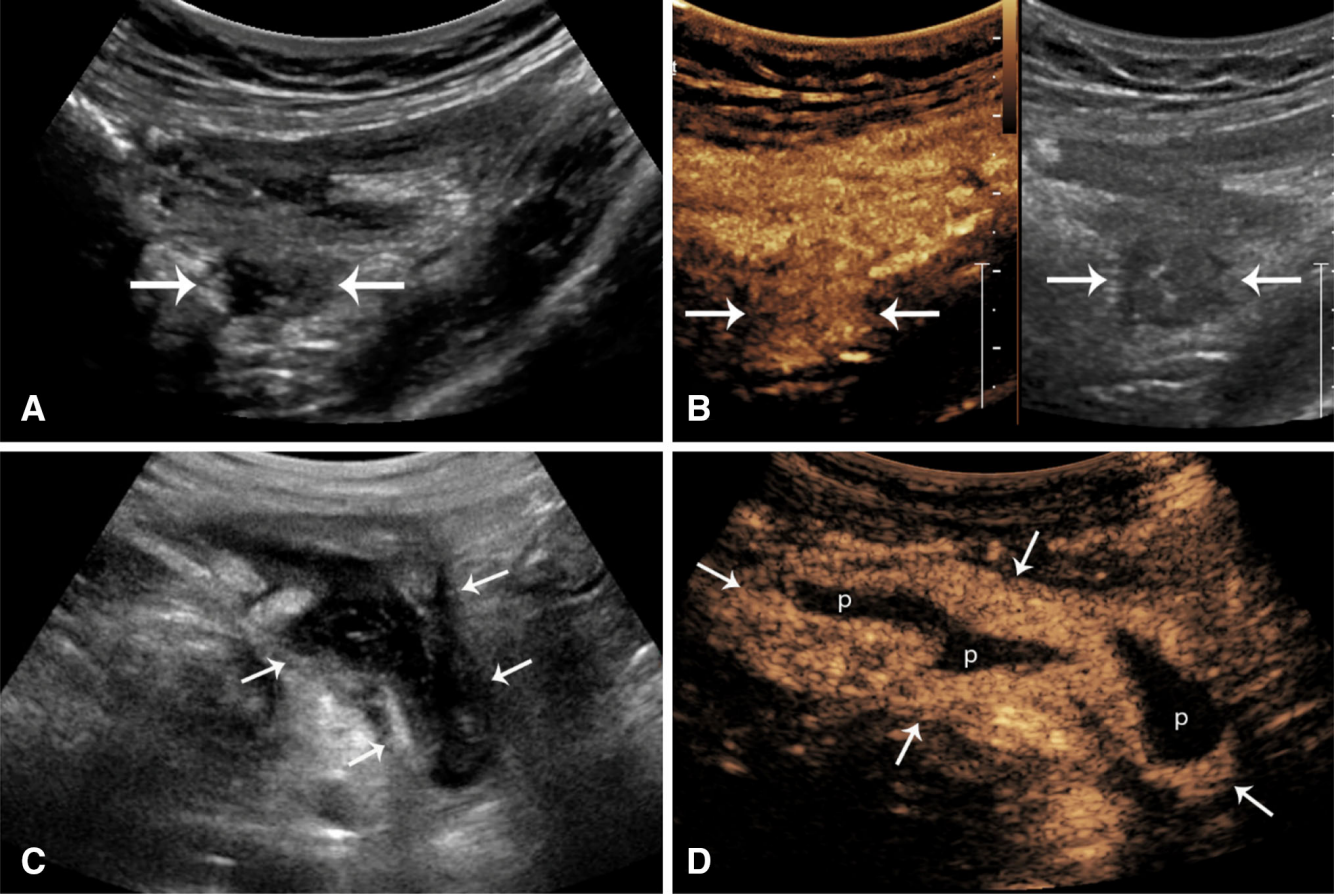
Resolution of inflammatory masses (arrows) with CEUS. **A** and **B**, inflammatory mass in an 81-year-old patient with CD. **A** Grayscale axial image in the pelvis shows a hypoechoic mass (arrows) deep to a loop of bowel. It is indeterminate in nature. **B** CEUS show homogenous hyperenhancement suggesting a mesenteric phlegmon (Supplementary Video 7). **C** and **D** A sagittal image of the right lower quadrant of a 46-year-old patient with CD shows a well-defined very black mass (arrows) R2-6 deep to the cecum at the pelvic brim. **D** CEUS shows a classic appearance of abscess with peripheral rim enhancement (arrows) and central avascular fluid/pus (P) (Supplementary Video 8)

Phlegmonous masses may show diffuse hyperenhancement reflecting acute inflammatory changes, demonstrated on color Doppler or CEUS (Fig. 7A, B, Supplementary Video 7). A phlegmon is generally treated conservatively with antibiotic therapy. An abscess, by comparison, will show regions of avascularity corresponding to pockets of pus with peripheral areas of enhancement, reflective of reactive inflammation, and the abscess wall [38] (Fig. 7C, D, Supplementary Video 8). Drainage of large abscesses is the general rule although small pockets of pus may also be treated conservatively. US/CEUS may be additionally helpful for guidance of percutaneous placement of drainage catheters and for monitoring response to therapy over time.

**Fig. 8:**
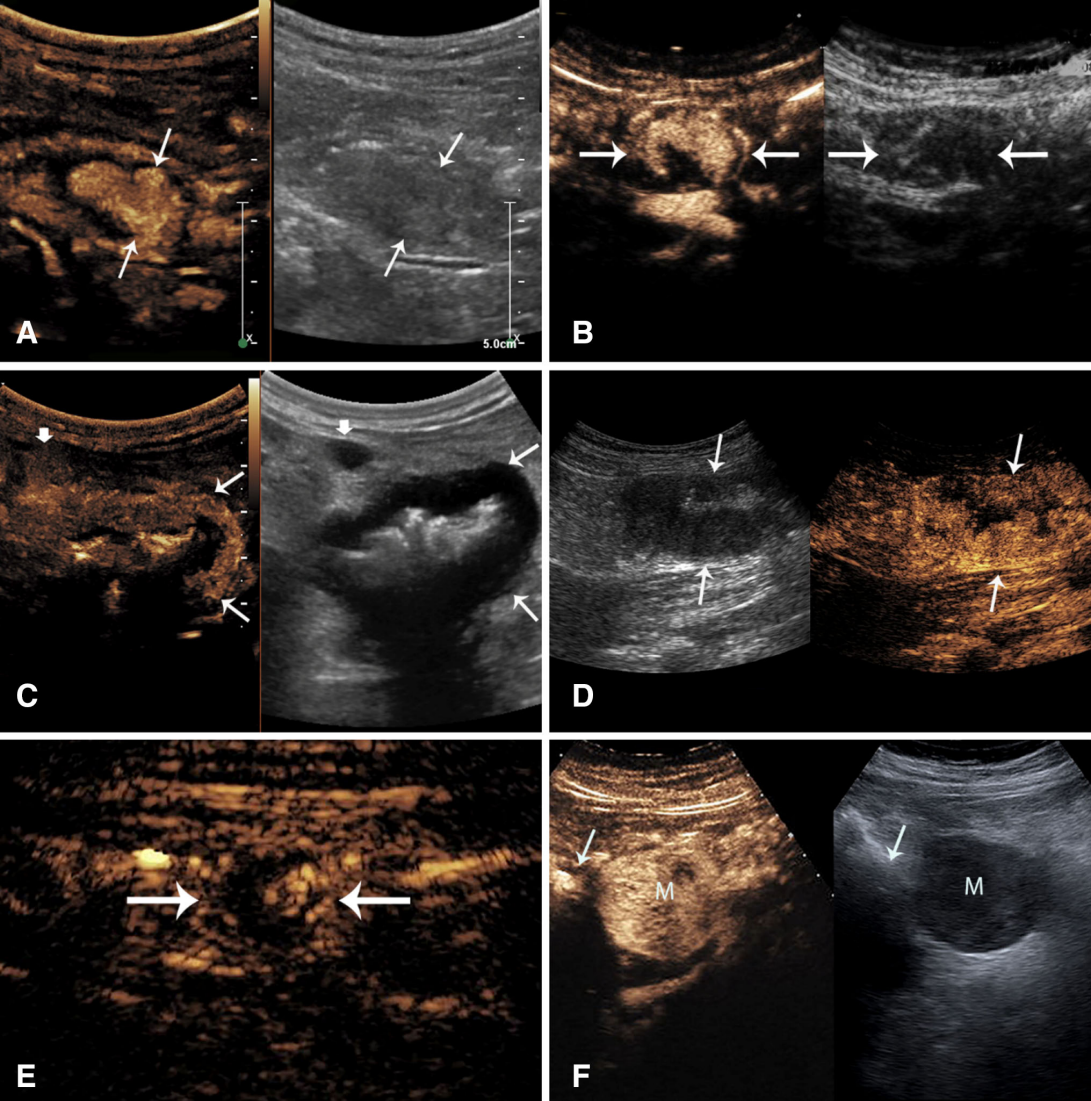
Bowel wall masses in six different patients. **A** Inflammatory polyp (arrows) in a 43-year-old patient with CD (Supplementary Video 9) shows as a vascular intraluminal mass in the NeoTI. **B** Carcinoid tumor of the small bowel (arrows) in a 52-year-old patient shows a mural black mass which is transiently hypervascular in the arterial phase (Supplementary Video 10). **C** Jejunal lymphoma (arrows) in a 68-year-old female with left flank pain and bloating and normal colonoscopy shows a thick walled segment with no wall layers, surrounding infiltrated fat and a big black lymph node (short arrow). The lumen shows aneurysmal dilation, highly suggestive of the correct diagnosis (Supplementary Video 11). **D** Descending colon adenocarcinoma in a 36-year-old woman. This apple-core lesion shows mural thickening of the bowel in a circumferential pattern with a narrowed lumen (Supplementary Video 12). **E** Small appendiceal carcinoid in a young woman with right lower quadrant pain, query IBS. The axial image of the appendix shows a fluid distended lumen and a small vascular polypoid mass (arrow) (Supplementary Video 13). **F** GIST tumor of the stomach in a 58-year-old patient presenting with epigastric pain shows a round enhancing mass (M) in the left upper quadrant, adjacent to the bowel (arrow). Its morphology is highly consistent with a GIST, adjacent to the stomach (Supplementary Video 14)

The study of inflammatory masses with US may be influenced by probe selection and placement. It includes the advantage of endovaginal scan in women, where the frequently involved pouch of Douglas is ideally evaluated and also the transperineal placement, in men and women, for confirmation of perianal abscess (Fig. 3). Inflammatory polyps may be suspected and confirmed as intraluminal vascular masses (Fig. 8A and Supplementary Video 9).

### Appendicitis and diverticulitis

Acute inflammatory conditions of the bowel include especially appendicitis and diverticulitis. At presentation, patients may have bowel perforation and/or an inflammatory mass, similar to those already shown with IBD. As previously described, these inflammatory masses can be further characterized with CEUS to differentiate phlegmon and abscess, as their clinical management would be different (Fig. 7).

Furthermore, CEUS may be a helpful adjuvant in early detection of wall ischemia, as a sign of gangrenous appendicitis that can be a precursor of perforation [37, 48]. In a study including 50 patients with suspected appendicitis, CEUS was 100% accurate in the identification of suppurative and gangrenous appendicitis when compare to surgical and histological findings [49].

Chronic diverticular disease may occasionally show colonic wall thickening and pseudomasses of uncertain etiology. This persistent hypoechoic wall thickening, in the absence of acute inflammatory changes, is most often related to familiar muscular hypertrophy.

### Epiploic appendagitis

This rare self-limiting inflammatory/ischemic process of the appendix epiploica of the colon may present as a hypo- and hyperechoic mass-like area next to the colonic wall at the site of patient’s pain that will enhance on CEUS as an inflammatory mass with a central area of non-enhancement that represents the central thrombosed pedicle, as occasionally seen on CT as the hyperdense central dot sign [50].

### Colitis and graft versus host disease (GVHD)

CEUS is only helpful for identification and grading of inflammation. However, it is not useful for differentiation of infectious colitis. CEUS has being described to be used only in a few instances where detection of wall necrosis or abscess may be a feature of condition, such as in cases of neutropenic enterocolitis [48, 51].

A few studies with a small number of patients also hypothesized that CEUS could detect microcirculatory changes of the bowel wall during evaluation of GVHD and to monitor therapy in this condition [48, 52].

### Intestinal ischemia

Bowel ischemia can be present due to bowel strangulation, thrombosis of the SMA, or non-occlusive mesenteric ischemia in patients with bowel obstruction. Patients with ischemia may have thin bowel walls as a result of loss of bowel wall tissue, vascularity, and muscular tone [39].

Studies have shown that lack of or decreased CEUS enhancement of the bowel wall is a hallmark for bowel ischemia with sensitivity of 85–94% and specificity of 100% [53]. In the case of bowel obstruction, this is seen most commonly in the least peristaltic segment and/or in the most dilated bowel segment [54].

In addition, CEUS has been used in other centers for adequate visualization of the visceral arteries. A European study combined CEUS with color and spectral Doppler allowing for an unequivocal diagnosis of visceral artery stenosis in patients with abdominal angina [55].

### Gastrointestinal tumors

A number of GI tumors can be incidentally encountered during bowel assessment including neuroendocrine tumors, gastrointestinal stromal tumors, adenocarcinoma, and lymphoma. Bowel wall neoplasms, regardless of their histology, generally show as hypoechoic masses on grayscale imaging which may grow with a polypoid intraluminal, mural, or serosal (Fig. 8). Their US study should always include careful assessment of the regional fatty mesentery and the lymph nodes.

As in other contrast imaging modalities, CEUS can delineate the tumor extent and vascularity very well. Some studies suggest that dynamic contrast-enhanced ultrasound with vascular recognition imaging software enables detection of micro-vessels and quantification of tumor perfusion. This has been used for assessment of treatment response and was correlated with survival [56].

CEUS can easily detect viable enhancing tumor versus areas of necrosis to guide target biopsy sampling and to minimize inadequate or insufficient sampling errors. This is particularly important in gastrointestinal stromal tumors (GIST) which can present as small or large masses within the peritoneal cavity, with or without an obvious attachment to a loop of bowel. They frequently show large areas of central necrosis or cystic degeneration, which is frequently suggestive of their diagnosis (Fig. 8F and Supplementary Video 14). On CEUS, GIST tumors show as a hypervascular mass in the arterial phase followed by slow wash-out during the portal venous phase [48]. In addition, in a small study of 24 patients with GIST, CEUS allowed for early prediction of tumor response to Imatinib treatment [57].

As compared with inflammation of the bowel which most often will involve a long segment, generally showing a stratified wall layering, most tumors show instead as a focal black mass, generally with complete wall layer destruction. Focal round masses of small and medium size identified incidentally during scans performed for IBD surveillance are often carcinoid (Fig. 8B, Supplementary Video 10 and 8E, Supplementary Video 13) or neuroendocrine tumors. They are highly vascular on CEUS and their wash-out correlates with their malignant potential [58].

### Lymphoma and adenocarcinoma

Lymphoma may show additionally an aneurysmal pattern of luminal expansion and lymph node involvement. (Figure 8C, Supplementary Video 11). Perienteric fat infiltration may be associated with all tumors.

Adenocarcinoma is seen most often in the large bowel although small bowel tumors are more prevalent in the population with IBD as compared with the general population (Fig. 8D, Supplementary Video 12).

**Fig. 9:**
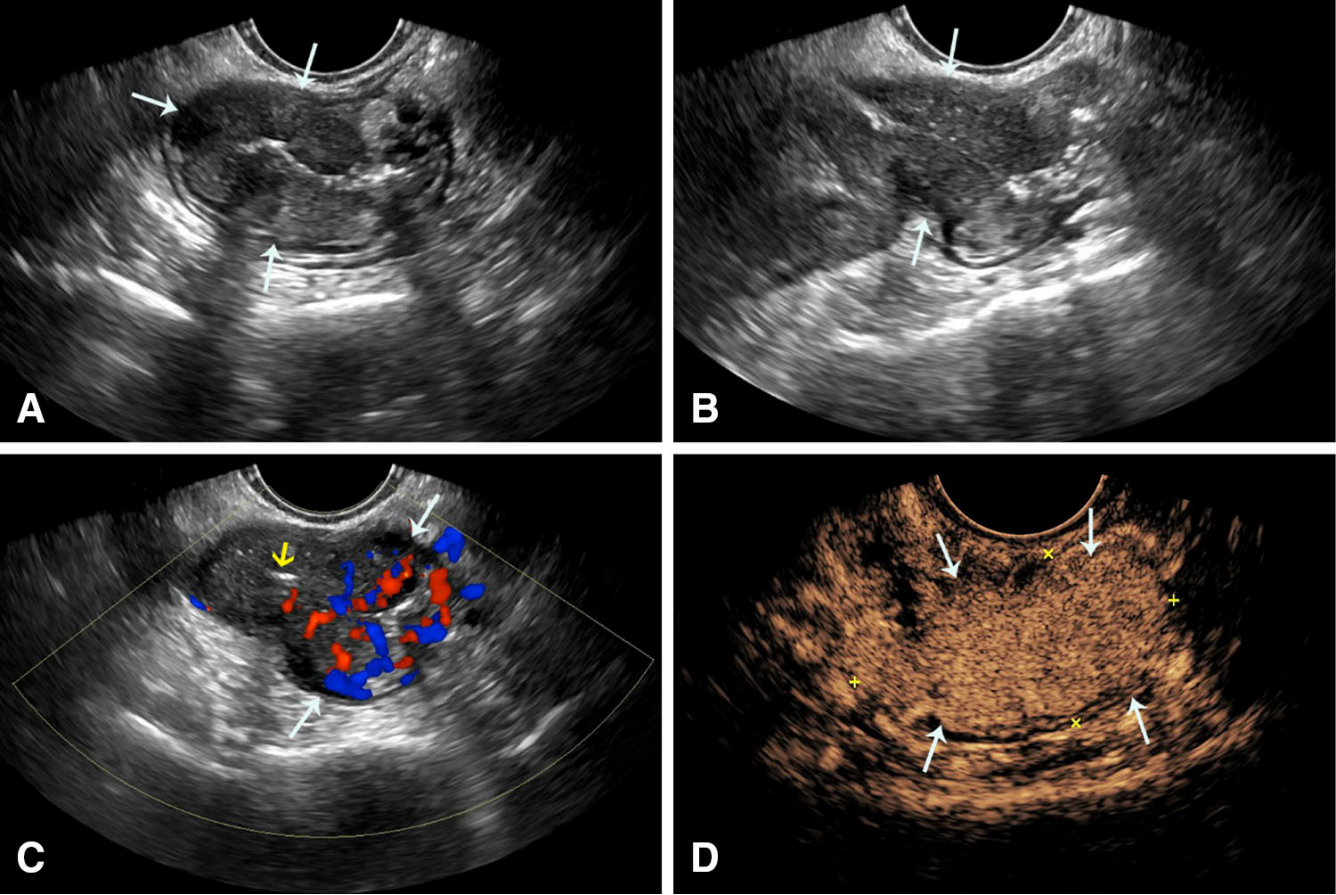
Rectal cancer on Endovaginal scan (EV) on a 37-year-old patient with rectal bleeding. **A** EV grayscale shows a lobulated hypoechoic asymmetric wall thickening/mass of the rectum (arrows). **B** Sagittal grayscale of the same abnormal segment shows an apple-core appearance (arrows). The lumen of the rectum shows as a white line on both figures **A**, **B**, and **C** (yellow arrow). Color Doppler shows the mass is hypervascular. **D** CEUS shows hyperenhancement of mass (yellow asterisk). WO is demonstrated in Supplementary Video 15

The exquisite resolution of endovaginal scans may improve evaluation of rectal cancer in women (Fig. 9, Supplementary Video 15) [59, 60].

## Conclusions—role of CEUS

CEUS may be utilized in evaluation of the bowel providing information about mural and mesenteric blood flow in a wide variety of situations. Similar to the use of intravascular contrast agents for CT and MR enterography, the addition of contrast agents to US will provide valuable information wherever blood flow knowledge would be helpful. However, it is in the evaluation of the population with IBD where the addition of CEUS has had its greatest clinical impact, substantially improving the role of ultrasound in the evaluation of the bowel. Although CDI is invaluable in bowel assessments, it has shortcomings which are easily overcome with CEUS. In addition, CEUS provides both subjective and objective quantification of mural blood flow. This is particularly important in the young population with IBD that requires frequent imaging surveillance and follow-up throughout the course of this chronic disease. Ultrasound and CEUS provide a non-invasive and accurate assessment of the classic features of CD and are highly sensitive for the detection and diagnosis of complication. In the future, CEUS of the bowel should be regarded as an essential component of standard care for imaging of the bowel in IBD.

## Electronic supplementary material

Below is the link to the electronic supplementary material.

Corresponding CEUS movie shows rapid transmural contrast enhancement that is sustained for a few seconds before slowly declining over time. Prominent mesenteric vessels are also enhanced. Supplementary material 1 (MP4 24,491 kb)

Same case as Figure 2. An endovaginal sweep through the thick segment of the terminal ileum shows a dilated fluid filled segment at the beginning of the movie before rapidly showing a long thick wall stricture with luminal apposition. There is a localized air containing perforation corresponding to figure 2C. At the end of the movie the stricture transition to normal looking bowel. Supplementary material 3 (MP4 1353 kb)

Same patient that in Figure 2, comb sign on CEUS. Supplementary material 2 (MP4 13604 kb)

Same case as figure 3 shows CEUS evaluation of abscess using a perianal approach. Supplementary material 4 (MP4 7467 kb)

CEUS generation of time intensity curves. Real time display of CEUS shows arrival of the microbubbles and a rise to a peak enhancement followed by a gradual decline toward the baseline over the next two minutes. Information from two ROI’s (red and yellow) generate the TIC shown at the bottom of the movie. This time intensity curve is a logarithmic display which optimally reflects the observer’s subjective evaluation of the enhancement. Supplementary material 5 (MP4 43,787 kb)

Indeterminate case, same patient as in figure 6. R2-5 CEUS shows transmural enhancement of the bowel wall. Supplementary material 6 (MP4 18,036 kb)

A vascular phlegmonous mass on an 81 year-old patient with CD, same as Figure 6, A and B. Supplementary material 7 (MP4 111,392 kb)

A periappendiceal abscess on a 46 year-old patient with CD, same as Figure 6, C and D. Supplementary material 8 (MP4 26,140 kb)

Inflammatory polyp of the ileum. CEUS shows a polypoid homogeneously hyperenhancing mass confirm on colonoscopy. Supplementary material 9 (MP4 12,063 kb)

Small bowel Carcinoid. CEUS shows hyperenhancing mildly lobulated mass within in the proximal ileum. Supplementary material 10 (MP4 4,996 kb)

Small bowel Lymphoma. CEUS shows enhancement of the abnormal black wall and adjacent abnormal lymph node. Supplementary material 11 (MP4 20,487 kb)

Descending colon adenocarcinoma. CEUS shows a multilobulated hyperenhancing mass with washout seen at the end of the movie. Supplementary material 12 (MP4 38,224 kb)

Appendiceal carcinoid. CEUS shows hypervascular enhancement of an intraluminal round mass within the fluid filled appendix. Supplementary material 13 (MP4 18,457 kb)

Gastric GIST. CEUS shows progressive hyperenhancement of the entire mass. Supplementary material 14 (MP4 10,252 kb)

Rectal Cancer. This axial movie, taken with an endovaginal probe, shows arrival of the microbubbles and uniform hyperenhancement of the mass. There is very rapid washout which is shown at the latter portion of the clip. This type of enhancement with rapid washout is frequently observed in malignant tumors of the bowel. Supplementary material 15 (MP4 30,224 kb)
